# Toward a Model of Personality Competencies Underlying Social and Emotional Skills: Insight From the Circumplex of Personality Metatraits

**DOI:** 10.3389/fpsyg.2021.711323

**Published:** 2021-11-11

**Authors:** Jan Cieciuch, Włodzimierz Strus

**Affiliations:** ^1^Institute of Psychology, Cardinal Stefan Wyszyński University in Warsaw, Warsaw, Poland; ^2^URPP Social Networks, University of Zurich, Zurich, Switzerland

**Keywords:** social and emotional skills, personality competence, Circumplex of Personality Metatraits, Self-motivation, Impulse control, Social responsibility, Assertiveness

## Abstract

In recent years, there has been a growing interest in social and emotional skills (SES) both in the scientific literature and in social practice. The paper presents an overview of the ways of understanding what SES are and the catalogs thereof. There are some attempts in the literature to organize these catalogs within the Big Five traits that for a long time was claimed to be the most sound model of basic orthogonal dimensions of personality. However, further research on personality structure revealed that two metatraits can be found above the Big Five traits. These two metatraits form the basis of the Two Factor Model of personality, which was later developed into the Circumplex of Personality Metatraits. It turned out that in certain aspects models based on metatraits have a greater theoretical potential than those based on the Big Five traits. The paper presents a proposal for describing SES from the perspective of the Circumplex of Personality Metatraits rather than the Big Five. In this framework, we distinguish the concept of personality competences that underlie and organize many specific SES and identify the core personality competencies on the basis of the Circumplex of Personality Metatraits model.

## Introduction

Social and emotional skills (SES) have, in recent years, been attracting growing attention from academics and practitioners. Such a shared interest in this topic among people from the worlds of science and practice, including education, economics, and politics, is an opportunity to gain scientific and practical benefits, especially a synergy effect in the understanding of the phenomenon and in transferring psychological knowledge to practice. At the same time, however, the diversity of approaches specific to each of these worlds generates many risks, including using the same concepts with different meanings or using different concepts with the same meaning, which may not only significantly reduce this potential synergy effect, but also reduce the chances of any progress in understanding the phenomenon and effectively transferring scientific knowledge to practice. The theoretical considerations and proposals presented in this paper aim to enhance the chance for these benefits and reduce the hazards.

The current state of knowledge on SES emerging at the intersection of science and practice can be described as follows. The concept of skills (and/or competencies) has been present in psychology for some time, particularly in the psychology of individual differences and educational psychology, but the ongoing intense growth of interest in this construct has not been due to the natural development of some theoretical models created as part of basic scientific research. This large increase in attention in the construct of skills was initiated outside academia – a demand was formulated in the fields of education, economics, and politics, to which academic psychology had to respond. In particular, the rapidly changing economic and social reality has led to a growing number of questions about the purpose of education. In a world of constant change and scientific or technological development, education can no longer mean equipping people with knowledge, because the increase in knowledge is so huge that it becomes outdated very quickly. As a result, both in psychological and pedagogical reflection and in official documents, the category of skills or competencies as desired outcomes of education began to appear. Moreover, education is no longer confined to the formal frame of school for children and youth, but extends throughout life in the paradigm of lifelong learning. In such a situation, various lists of these skills as desirable characteristics began to be formulated in a number of areas outside academia and it turned out that a large part of them concerns broadly understood social and emotional functioning. Academic psychology was thus faced with the challenge of scientifically elaborating both the construct of SES itself as well as a list of these skills that would be consistent with current knowledge in the field of psychology of personality and individual differences. The first answers from psychology have already been given in the literature and were as one would expect. Namely, the focus was put on the taxonomy of SES in the Big Five framework (Goldberg, 1990; McCrae and Costa, 2003), where the richness of many personality traits is organized in five main orthogonal personality dimensions (neuroticism/emotional stability, extraversion, openness to experience/intellect, agreeableness, conscientiousness), and in some models in the Big Five framework, each of these dimensions is composed of several facets. Using the Big Five to describe SES was understandable and, one might say, quite obvious. After all, if in psychology there is a set of personality variables that need to be organized, the Big Five usually seems to be the natural approach or at least the point of departure. And so, it was the case this time. The richness of many SES differentiated in several models has been sorted into five domains corresponding to five personality traits as we describe in detail in the subsequent part of the paper.

We argue, however, that such an approach to SES, although natural and convincing at first glance, is nevertheless nonoptimal, because, apart from a simple classification, it in fact does not offer any significant insight into the nature of SES and, therefore, does not allow for the optimization of their formation in education. Moreover, we do not conclude our contribution with such a critical part. Quite the contrary – we suggest an alternative view. Its essence is: (1) adoption of the framework of the Two Factor Model of personality (TFM; review in Cieciuch and Strus, 2017), extended to the Circumplex of Personality Metatraits (CPM; Strus et al., 2014) – as the basis for understanding and classification of SES in place of the previously adopted Big Five framework, and (2) introduction of the concept of *personality competence*, distinguished from SES and being a basis for organizing and developing many specific skills.

More specifically, in this paper, we formulate and justify, based on a thorough literature review, the following claims:

1. Many approaches to skills agree on defining skills as (1) malleable and (2) positive (desirable) characteristics of a person.
2. Skills should be differentiated from traits and abilities, and we propose a way to do it.
3. The models that appear in the literature aimed at integrating skills (usually based on the Big Five) have some limitations.
4. Switching the foundations of SES models from the Big Five to CPM enables the identified problems with SES models to be resolved.
5. Specific social, emotional, and motivational skills (SEMS) are shaped on the basis of four personality competencies, differentiated in CPM.
6. The way in which we propose organizing SES can, in the future, also be expanded to the cognitive domain that can lead to a complex model of all psychological skills and competencies.

## Defining Skills

### Common Characteristics of Skills

The concept of skills usually emerges while responding to the question of which human features are responsible for one’s effective coping with life and well-being. This general question is made more specific with respect to different contexts concerning both individuals and society. Regarding individuals, these contexts include, for example: a school context (what makes students successfully cope with learning), a vocational context (what makes people successful in the labor market), an interpersonal context (what makes people function well in various types of interpersonal interactions), or a general personal development context (what accounts for a person’s self-realization and happiness). In relation to the functioning of societies, this can be, for example, a civic context (what psychological characteristics account for the good functioning of society) or an economic context (what psychological characteristics account for sustainable economic development). Thus, the concept of skills emerges when scientific psychological knowledge is applied to improve the quality of life of people and societies.

When used in this way, the concept flickers with the meanings originating from various contexts and fields of use. At the same time, however, there are two characteristics of skills that are invariant across contexts and seem to apply to all of them.

Firstly, skills are associated with positive valence. Thus, they are desirable characteristics, which are better to have than not to have. The search for positive human characteristics is especially underlined in the positive psychology framework. Instead of a focus on deficiencies and pathologies of functioning, positive psychology has made calls to focus on positive characteristics and ways to enhance them (Eccles and Gootman, 2002; Peterson and Seligman, 2004; Bowers et al., 2015). Skills are assessed positively because they are associated with a variety of positive outcomes. Sometimes, these are outcomes related to a particular area of life, such as work, and sometimes to well-being in general, as the most universal and most positive outcome (reviews of the association of skills with positive outcomes in: Borghans et al., 2008a; Kautz et al., 2014; Kankaraš, 2017; Chernyshenko et al., 2018).

Secondly, skills are understood as being malleable, that is, they can be shaped in the process of various interactions and education – formal, informal, lifelong education, self-education, etc. It is worth noting that considering skills as malleable is one of the defining features reflecting the origins of interest in this construct. Generally, in psychology, the question of whether a given characteristic is stable or can be changed and shaped is an important one. Answers to this question are ultimately provided in the course of long-term empirical research. Moreover, these answers are not usually conclusive in the zero-one sense. However, since much of the interest in the construct of skills is the result of demand from the world of practice focused on the improvement of people’s and society’s quality of life, this malleability has from the beginning been expressed explicitly or assumed implicitly. After all, if skills are to be the goal of education, they must be malleable.

### What Are Social and Emotional Skills and What Are They Not?

Psychological variables that determine career and life success have been identified in the literature and then usually divided into two types of skills: cognitive and noncognitive. The first studies on this topic appeared in economics, and the subject of interest was the relationship between professional success (usually measured by earnings) and education (Becker, 1964; Ben-Porath, 1967). These first papers introduced the construct of *cognitive ability*. In contemporary psychology, abilities are usually distinguished from skills in such a sense that abilities are just a potential, while skills are acquired and developed intentionally through experience and practice (Riggio, 2017). In the past, this differentiation was, however, not always made clear enough and the same cognitive variables measured by various intelligence or achievement tests were sometimes labeled as *abilities* (Kuncel et al., 2004) and sometimes as *skills* (Burks et al., 2009). After decades of research, they have become a generally accepted predictor of successful vocational functioning.

Once the role of cognitive skills in achieving life success was established in the literature, research began to emerge demonstrating the significance of other skills than the cognitive ones in achieving this goal. They were usually labeled as *noncognitive skills* (Heckman and Rubinstein, 2001; Heckman et al., 2006; Thiel and Thomsen, 2013; Kautz et al., 2014) and referred to broadly understood personality characteristics, including personality traits (Borghans et al., 2008b; Almlund et al., 2011; Becker et al., 2012). In this context, other terms also appear: the term *soft skills* (Heckman and Kautz, 2012; Balcar, 2014; Koch et al., 2015; Lippman et al., 2015) and more and more often, especially in psychological literature – the term *socio-emotional skills* (Koch et al., 2015; Kankaraš, 2017). Noncognitive skills have typically been considered as comprising a larger number of constructs with a less well-described structure than cognitive skills (Heckman and Rubinstein, 2001).

The distinction between cognitive and noncognitive skills, while intuitively understandable, is nonetheless quite problematic from the psychology of personality point of view (Duckworth and Yeager, 2015). The concept of noncognitive skills has in fact been criticized in psychology for as long as it has been in use (Messick, 1979). Of course, one can identify prototypical skills for both domains (e.g., patience as a noncognitive skill and reading speed as a cognitive skill). However, the disadvantage of such a distinction is the problematic exclusion of cognitive aspects from given noncognitive skills. And yet, in fact, most or maybe even all skills possess an aspect of some processing of information, which is exactly the cognitive aspect (Duckworth and Yeager, 2015). On the other side, it also seems to be impossible to separate cognitive skills from noncognitive elements. Interestingly, even Wechsler himself wrote in 1943 regarding intelligence: “in addition to intellective there are also definite nonintellective factors which determine intelligent behavior” (Wechsler, 1943, p. 103). Today, the impact of noncognitive skills on the results of tests measuring cognitive skills is quite obvious (Borghans et al., 2011).

Whereas the distinction between cognitive skills and noncognitive skills is difficult to carry out systematically and, in general, lacks important practical consequences, the distinction between skills and traits is essential from both a theoretical and practical point of view (Soto et al., 2020). According to Soto et al. (2020), in the personality domain, the difference between skills (or, more precisely, SES) and traits (or, more precisely, the Big Five personality traits) can be described using the pair of concepts of capacity vs. tendency. SES relate to a domain-specific *capacity* for doing something, while personality traits relate to a cross-situational *tendency* for doing something. This relatively precise distinction allowed Soto et al. (2020) to use knowledge accumulated in the Big Five research tradition to group and describe SES. While it is a step in the right direction, this proposal does raise some problems, which will be discussed in the following paragraphs of this paper. Still, the distinction between skill-like and trait-like constructs is itself valuable and worth applying to the cognitive domain as well. It can help to differentiate between cognitive skills (skill-like construct) and abilities (trait-like construct). However, there are also two important differences between trait-like constructs from the personality domain and cognitive domain. The first is that trait-like constructs in the personality domain are a tendency, while in the cognitive domain, they are an ability. The second is that the trait-like constructs from the personality domain are usually bipolar, that is, both poles of a given aspect have their own psychological characteristics; one pole is not merely the absence of the characteristics of the other pole (e.g., introversion is not merely the absence of extraversion). In contrast, the trait-like constructs from the cognitive domain are unipolar, that is, one pole is defined and the other pole represents the lack of a given characteristic (e.g., low intelligence means only a lack of high intelligence).

The above differentiations are summarized in Table 1. In this view, the constructs that differ the most are intelligence (cognitive ability) and SES (noncognitive capacity). From the point of view of personality psychology, it is also crucial to distinguish between a skill and a trait, since the former is a possible object of intervention and education, while the latter is not or only to a small extent (depending on how the traits are defined). In other words, skills are malleable, while traits are rather stable. Regarding the distinction between cognitive and noncognitive skills, it can be assumed after Messick (1979) that cognitive means not only cognitive, and noncognitive does not imply the absence of cognitive. While at the level of trait-like constructs, this differentiation can still be clearly maintained; at the level of skills, the boundaries become blurred. This is because in the case of skills, it is rather a distinction between cognitive and noncognitive *aspects* of a given skill. Of course, for some skills, the cognitive or noncognitive aspect may be rather small and can be omitted. In this paper, we focus on the SES in which the cognitive aspect is not very significant. However, after presenting our proposal, we show the usefulness of expanding it to the cognitive domain.

**Table 1:** Different types of characteristics fostering success and well-being.

Characteristic	Cognitive	Noncognitive
Trait-like	*Ability* e.g., Intelligence	*Tendency* e.g., The Big Five traits
Skill-like	*Capacity*	
	Cognitive skills	Social and emotional skills

## Catalogs of Social and Emotional Skills or Skills with Social and Emotional Content

In the literature, there are many catalogs of skills, sometimes referred to by different names (reviewed in: Berg et al., 2017), and they can be generally divided into three types, based on whether they originate from the basic scientific research or the world of practice. One type is generated by researchers within academia to describe individual differences relevant from a theoretical point of view. The second type is generated by practitioners, policymakers, or stakeholders, and its essence is to identify skills relevant from the perspective of the demand from the changing world, economy, and new social challenges. The third type of catalog is generated in collaboration between academics and practitioners, that is, the skills desired from a practical point of view are identified, but at the same time, they are subjected to intensive scientific research. Finally, there are some attempts to integrate the distinguished catalogs of skills. It should be borne in mind that, in all cases, there are also catalogs that are not called catalogs of skills by their authors, but the characteristics of the distinguished constructs meet the definitional criteria of skills. The following paragraphs will discuss the best-known representatives of each type and then present our model, located in the third (synthesizing) type but overcoming the problems of current proposals.

### Catalogs Developed Within Basic Research

Examples of the first type of catalog are proposals that use terms other than skills, but which are *de facto* lists of skills in the sense presented above. These are: character strengths catalog of Peterson and Seligman (2004) and emotional competencies catalog of Saarni (2000). List of character strengths of Peterson and Seligman (2004) appears in various reviews of SES (Berg et al., 2017; Soto et al., 2020) because character strengths possess both of the properties of skills identified above: They are positive and malleable (Peterson and Seligman, 2004). Peterson and Seligman (2004), through their concept of character strengths, draw the attention of psychologists to those aspects of human functioning that enable a person to develop, grow, and to have a good, happy life, or in another words – the aspects leading to, constituting, and explaining eudaimonistically understood well-being. They look for these attributes not only in the scientific psychological literature, but also in the classical philosophical and religious literature as well. As a result, they built a list of qualities that have been valued by moral philosophers, religious thinkers, and lay people over the centuries. The list consists of 24 character strengths assigned to six virtues: (1) Wisdom (creativity, curiosity, open-mindedness, love of learning, and perspective), (2) Courage (bravery, persistence, integrity, and vitality), (3) Humanity (love, kindness, and social intelligence), (4) Justice (citizenship, fairness, and leadership), (5) Temperance (forgiveness, humility, prudence, and self-regulation), and (6) Transcendence (appreciation of beauty, gratitude, hope, humor, and spirituality). However, based on empirical research, these 24 character strengths are usually grouped into five domains corresponding to the Big Five traits (review in Najderska and Cieciuch, 2018).

Saarni (2000), on the other hand, focused on what is necessary for navigating the demands of the social context and distinguished the following skills (named by her as competencies): (a) awareness of one’s emotional state, (b) discerning others’ emotions, (c) using the vocabulary of emotion, (d) empathic and sympathetic involvement in others’ emotional experiences, (e) understanding the difference between inner emotional state and outer expression, (f) adaptive coping with aversive or distressing emotions, (g) understanding the influence of emotions on the relationship, and (h) emotional self-efficacy. Saarni (2000) emphasizes that the distinguished skills are not independent – they are interrelated and condition each other. Such linkages beg the question of whether there are any more fundamental dimensions underlying the distinguished skills.

### Catalogs Developed Outside Academia

The catalogs of the second type are developed outside academia, although scholars are often involved in their development. Their core is the identification of those skills, the acquisition of which increases, on the one hand, the chances for professional success and personal well-being, and on the other hand, economic development or good functioning of society. It happens that they do not have a personal authorship, because their authors are organizations or institutions. Interestingly, they are usually not introduced in the classical scholarly circuit – a system of publication in peer-reviewed journals that over the years turns into an archive that can be used to trace the evolution of a construct. As a consequence, it is sometimes difficult to reconstruct such an evolution, and it also happens to be the case that the understanding of a given construct is simply changed by a given organization or by another organization that in one way or another builds on the previous one. This is the case with so-called 21st century skills. It is a kind of widespread approach that draws attention to the need to change the thinking about what skills will be needed for personal and professional success in the 21st century (Trilling and Fadel, 2009). The movement was initially closely associated with the Partnership for 21st Century Skills, founded in 2002 as a nonprofit organization gathering members from the fields of business, education, and politics. This association distinguished three groups of 21st century skills: (1) learning and innovation skills, (2) information, media, and technology skills, and (3) life and career skills. Today, however, the ideas of the Partnership for 21st Century Skills are being pursued by Battelle for Kids, an American not-for-profit organization whose mission is to promote the teaching of 21st century skills. Battelle for Kids was established in 2001, and in 2018, the organization was joined by the Partnership for 21st Century Skills (according to information available at https://www.battelleforkids.org/about-us on 04/02/2021). The resources currently available in the public domain offer slightly varying lists of 21st century skills compiled by Battelle for Kids, Partnership for 21st Century Skills or their members. Trilling and Fadel (2009), co-authors of this approach as part of the Partnership for 21st Century Skills, distinguish even more specific skills within the above-mentioned types of skills. According to them, learning and innovation skills include the following: (a) critical thinking and problem solving, (b) communication and collaboration, and (c) creativity and innovation. Information, media, and technology skills – from the main classification – become, in their approach, specific skills from the area of digital literacy skills. Life and career skills, meanwhile, include the following: (a) flexibility and adaptability, (b) initiative and self-direction, (c) social and cross-cultural interaction, (d) productivity and accountability, and (e) leadership and responsibility. However, other detailed suggestions can also be found on the websites of Battelle for Kids,^1^ which indicates that the essence of this proposal is a general approach rather than detailed catalogs.

The construct of 21st century skills and the list thereof were further refined by the National Research Council by establishing the Committee on defining deeper learning and 21st century skills under the leadership of Pellegrino and Hilton (2013). This approach adopts the concept of *competence* as a term organizing various skills and at the same time linked to a person’s knowledge and attitudes (Pellegrino and Hilton, 2013). Proposal of Pellegrino and Hilton (2013) to organize skills is presented in the next paragraph.

Using the term of competencies in the meaning indicated above originated from the OECD (2005) and was adopted by the European Commission in the Council Recommendation of 22 May 2018 on key competences for lifelong learning (The Council of the European Union, 2018). It employs the category of key competence defined as a combination of knowledge, skills, and attitudes. The Council of the European Union (2018) identifies eight key competencies “necessary for employability, personal fulfilment and health, active, and responsible citizenship and social inclusion”: (1) literacy competence, (2) multilingual competence, (3) mathematical competence and competence in science, technology, and engineering, (4) digital competence, (5) personal, social, and learning to learn competence, (6) citizenship competence, (7) entrepreneurship competence, and (8) cultural awareness and expression competence.

### Catalogs Developed at the Meeting Point of Basic and Applied Research

Another prominent example of a construct introduced by an organization into the public, but in this case also much more into the scholarly circuit, is the construct of social and emotional learning (SEL) and the organization is the Collaborative for Academic, Social, and Emotional Learning (CASEL). The organization was founded in 1994 by, among others, Goleman (1996), one of the main propagators of the concept of emotional intelligence. The goal of the CASEL was establishing and promoting SEL as a crucial part of education. The construct of SEL is present in papers published in mainstream scientific psychology journals (e.g., Durlak et al., 2011; Domitrovich et al., 2017). Here, the focus is directly on the category of learning, and thus, SEL is defined as the process through which people (both children and adults) acquire and effectively apply the knowledge, attitudes, and skills, collectively referred to as competencies (Jagers et al., 2019). From the perspective of SES, what is crucial is the defining purpose of this process of learning and that is precisely the acquisition of core social and emotional competencies. In the CASEL proposal, these core social and emotional competencies are as follows: (1) self-awareness, (2) self-management, (3) social awareness, (4) relationship skills, and (5) responsible decision-making (CASEL - Collaborative for Academic, Social, and Emotional Learning, 2003; Jagers et al., 2019).

**Figure 1:**
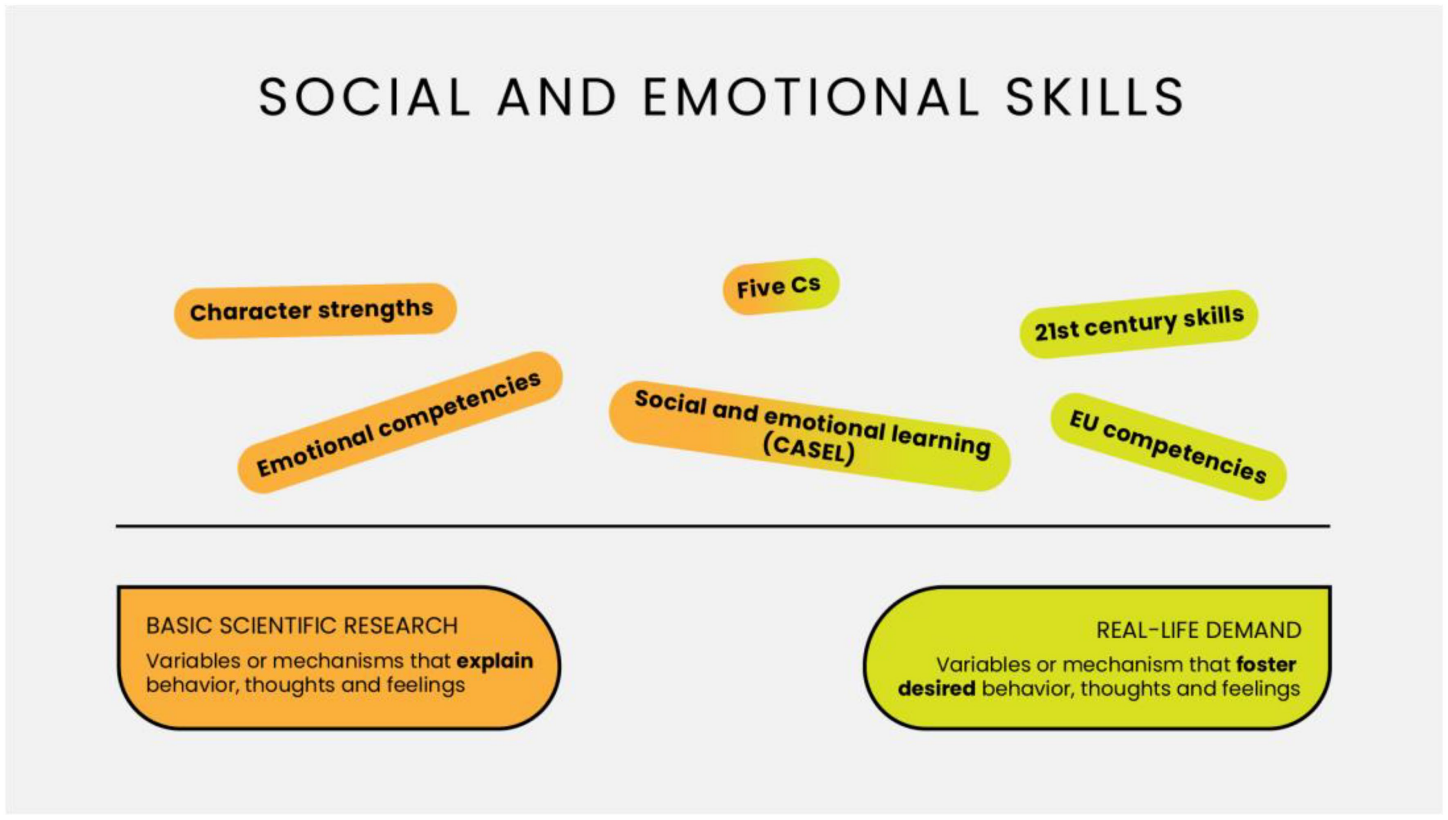
The dual genesis of various types of skills catalogs.

Another list of positive, malleable characteristics that emerged from a collaboration of practitioners, policy makers, and researchers is the so-called Five Cs model proposed by Lerner (review in: Geldhof et al., 2015). It was proposed within the positive youth development perspective, which proposes an education focused on developing positive qualities instead of an education focused on making up deficits (Lerner, 2015). The Five Cs that group the positive qualities are as follows: competence, confidence, character, caring, and connection. The list groups together characteristics similar to the character strengths proposed by Peterson and Seligman (2004) discussed earlier, but unlike the latter, it is oriented toward identifying the purposes of school education and from the outset has been introduced into both research and educational practice (Phelps et al., 2009; Bowers et al., 2010; Lerner and Lerner, 2013). Therefore, it is situated at the meeting point of basic and applied research. Figure 1 graphically depicts the dual genesis of various catalogs of skills.

## Prior Attempts at Organizing Social and Emotional Skills

### A Map of All Skills

In the literature, there have been attempts to integrate several catalogs of skills and similar constructs and develop a kind of map of all skills, including both cognitive skills and SES. Each time, the starting point is some well-established model in the psychology of individual differences, which becomes a kind of reference system for organizing the distinguished skills.

For example, the National Research Council (Pellegrino and Hilton, 2013), in order to structure skills, adopted two well-established taxonomies of individual differences. For cognitive skills, it was the “three stratum” hierarchical model of intelligence with the general cognitive ability factor at the top, eight second-order abilities (factors) at the second stratum, and more narrowly defined abilities at the third stratum (Carroll, 1993). The Big Five framework (Goldberg, 1992; McCrae and Costa, 2003), on the other hand, was adopted for noncognitive skills. The various detailed lists of 21st century skills are sorted by the National Research Council (Pellegrino and Hilton, 2013) into three main groups labeled as competencies: (1) cognitive, (2) intrapersonal, and (3) interpersonal. Within each group, three clusters were distinguished, and then, each of these clusters was assigned to one of the Big Five traits (clusters from intrapersonal and interpersonal groups) or to one of the first three factors of intelligence (clusters from cognitive group) at the second stratum of the “three stratum” model (Carroll, 1993): fluid intelligence, crystallized intelligence, general memory, and learning.

**Figure 2:**
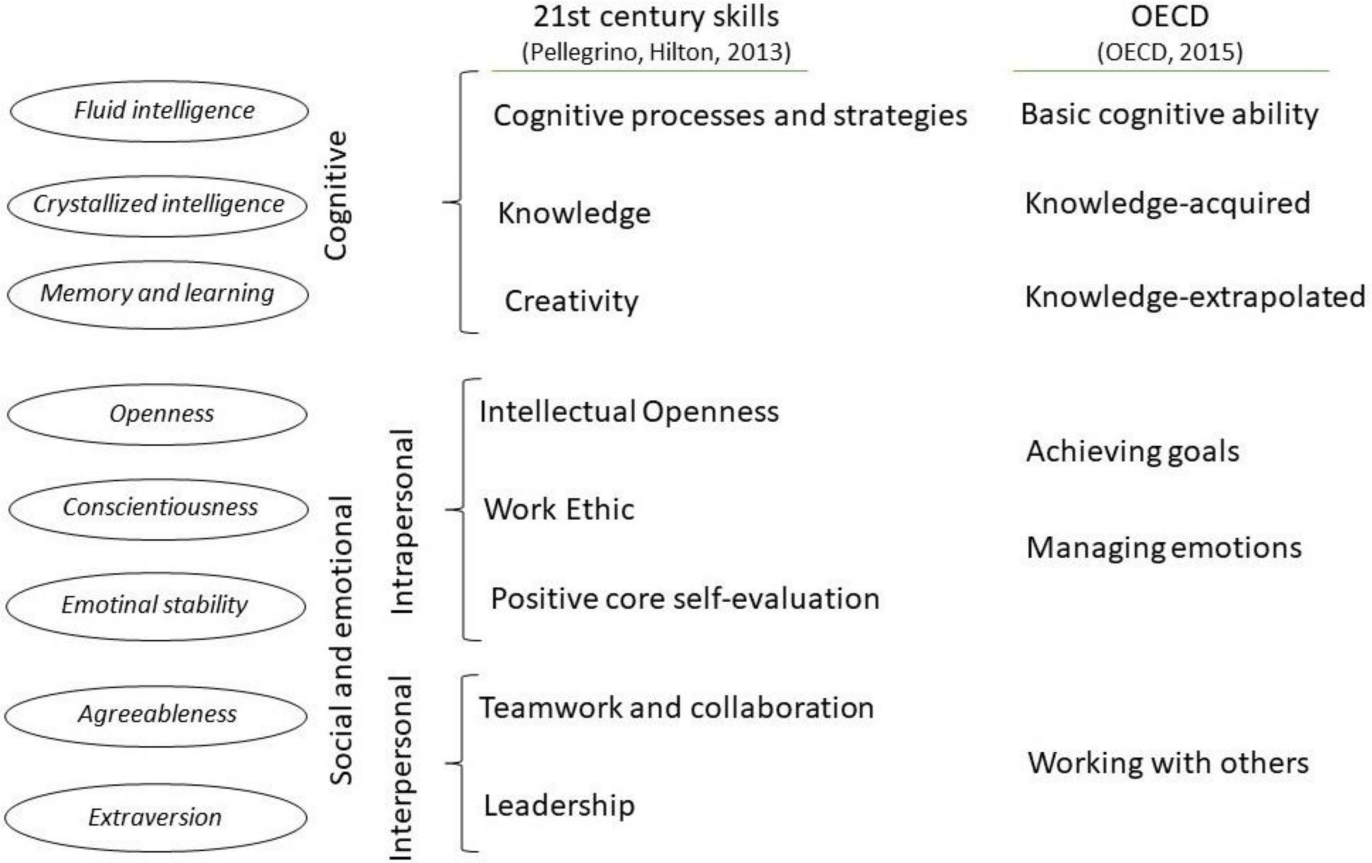
Proposals of structuring skills according to the National Research Council (Pellegrino and Hilton, 2013) and the OECD (2015).

The OECD (2015) also adopted a similar categorization of skills into (1) cognitive and (2) SES and distinguished three clusters in each of these two groups. Figure 2 depicts propositions of such general maps of skills by the National Research Council (Pellegrino and Hilton, 2013) and the OECD (2015) along with the corresponding constructs from the hierarchical model of intelligence and the Big Five.

This paper deals with SES, so they are the ones we will focus on, and we will come back to the whole map after presenting our theoretical proposal.

### Taxonomy of Social and Emotional Skills

The attempts to integrate different lists of SES have, to date, generally been made in the Big Five framework (Pellegrino and Hilton, 2013; Kautz et al., 2014; De Fruyt et al., 2015; John and De Fruyt, 2015; Kankaraš, 2017; Chernyshenko et al., 2018; Kankaraš et al., 2019; Kankaraš and Suarez-Alvarez, 2019; Soto et al., 2020, 2021). The literature indicated above presents proposals of integrating various lists of SES within the framework of the Big Five, also taking into account lower-order traits (for a review of structural models of personality, see: Strus and Cieciuch, 2014). Table 2 summarizes these attempts by taking into account the most synthetic ones, that is, the theoretical and empirical synthesis in the OECD study (Kankaraš and Suarez-Alvarez, 2019) and the proposal of Soto et al. (2020, 2021). The work on organizing the skills in both of these approaches followed three similar steps. As a first step, both Soto (Soto et al., 2020) and researchers under the OECD (John and De Fruyt, 2015; OECD, 2015) sorted out the various existing lists of SES by assigning the distinguished skills to the Big Five domains. Since the Big Five proved to be effective in integrating catalogs of SES created outside this paradigm as well, in the second step, Big Five was no longer used to organize other lists of SES that more or less matched the Big Five domains, but to find SES within facets distinguished in several Big Five models. In the research conducted under the OECD (Kankaraš and Suarez-Alvarez, 2019), the second step consisted of selecting skills from those facets that meet the following criteria: (a) predictive value, (b) malleability, (c) appropriateness for children and adolescents, (d) possible to measure in a comparable way across cultures, (e) relevant for the future world, and (f) already well researched (Kankaraš and Suarez-Alvarez, 2019). Soto et al. (2021), on the other hand, selected from various hierarchical Big Five models those facets that correspond to skills they defined as capacities used to maintain social relationships, regulate emotions, and manage goal- and learning-directed behaviors. It should be added here that Soto et al. (2021) expand the name of “social and emotional” (SE) skills to social, emotional, and behavioral (SEB) skills to better reflect the scope of the skills domain, as well as to distinguish the acronym describing SES from SES for socioeconomic status, which is often used in the literature. In addition, Soto et al. (2020) identified prototypical SES within each domain, thus emphasizing that the lists are not closed. Table 2 presents specific SES identified in both approaches assigned to the five personality domains. Moreover, the third step in both proposals involved developing an operationalization of the identified SES. The OECD researchers (Kankaraš and Suarez-Alvarez, 2019) largely used items from the International Personality Item Pool resources (Goldberg et al., 2006). In contrast, Soto et al. (2021) created items in such a way as to distinguish capacity (definitional for skills) from tendency (definitional for traits).

**Table 2:** Specific social and emotional skills located within Big Five domains in two approaches.

The Big Five	The proposal of Soto et al. (2020, 2021)	The proposal of OECD (Kankaraš and Suarez-Alvarez, 2019)
Conscientiousness	Self-management	Task performance
	– **Task management**	– Self-control
	– Time management	– Responsibility
	– Detail management	– Persistence
	– Organizational skill	
	– Responsibility management	
	– Capacity for consistency	
	– **Goal regulation**	
	– Rule-following skill	
	– Decision-making skill	
Extraversion	Social engagement	Engagement with others
	– **Leadership skill**	– Sociability
	– Persuasive skill	– Assertiveness
	– **Conversational skill**	– Energy
	– Expressive skill	
Agreeableness	Cooperation	Collaboration
	– Teamwork skill	– Empathy
	– Capacity for trust	– Trust
	– **Perspective-taking skill**	– Cooperation
	– **Capacity for social warmth**	
Openness	Innovation	Open-mindedness
	– **Abstract thinking skill**	– Tolerance
	– Creative skill	– Curiosity
	– **Artistic skill**	– Creativity
	– Cultural competence	
Emotional stability	Emotional resilience	Emotion regulation
	– **Stress regulation**	– Stress resistance
	– Capacity for optimism	– Optimism
	– **Anger management**	– Emotional control
	– Confidence regulation	
Blends or additional traits	– Energy regulation (blend of Self-management with Social engagement)	Additional indices– Achievement motivation– Self-efficacy
	– Ethical competence (blend of Self-management with Cooperation)	
	– Information processing skill (blend of Self-management with Innovation)	
	– Impulse regulation (blend of Self-management with Emotional resilience)	

In bold – examples of prototypical skills from Soto et al. (2020).

Both proposals led to a fairly similar list of variables, with Soto’s proposal (Soto et al., 2020, 2021) systematically differentiating between skills and traits in addition to the catalog of SES in the Big Five framework, as well as deriving implications of this differentiation for measurement. Both proposals, while interesting in their holistic approach, nevertheless inherit the concerns and problems associated with the Big Five, as will be discussed below.

## Problems with Past Proposals to Organize Social and Emotional Skills Under the Big Five Framework

Attempts to organize personality variables into Big Five domains are a fairly natural and frequently used approach when in a given personality sphere many constructs are differentiated with unclear relations between them. Such an approach was applied, for instance, to 24 character strengths, which, as it turned out, can also be clustered into factors similar to the Big Five (review in Najderska and Cieciuch, 2018). This was also applied to attempts at describing personality disorders in the dimensional approach proposed by DSM-5 (American Psychiatric Association, 2013) and ICD-11 (World Health Organization, 2020), which also distinguish five domains analogous to the Big Five. A similar approach has also been applied to organizing the traits characterizing specific categories of personality disorders (cf. Bagby and Widiger, 2018). This approach transforms an unorganized set of characteristics into an ordered structure, which is of significant added value, especially at the initial stages of research on a given phenomenon. At the same time, however, this approach has its limitations, some of which are inherited from the Big Five model itself.

Interestingly, the application of the Big Five to describe other spheres of personality intensified when basic research on personality structure led to the conclusion that the Big Five cannot be treated as a final model of basic personality trait structure any longer. In psychology, it rarely happens that one dominating model is completely replaced by another. Rather, competing models appear, which in some way improve or complement those previously dominated and considered most accurate. They all continue to coexist in the scholarly circuit, and the initial model is no longer the only point of reference. This is the situation of the Big Five. Its potential to organize the area of personality traits is unquestionable, which is shown in a vast amount of literature. At the same time, however, if there are new personality spheres to be described using the basic dimensions of personality, then the Big Five is no longer the only candidate worth considering, and one could even argue that it is not the best one. This is because, on the one hand, models have emerged that propose a different number of basic dimensions that are at least equally well-supported (especially the HEXACO model; Ashton and Lee, 2007, 2020), and on the other hand, there are other structural proposals that offer an integration of various models of personality and emotional and social functioning in at least as broad a manner as the Big Five models (especially the CPM; Strus et al., 2014; Strus and Cieciuch, 2021).

Strus et al. (2014) have distinguished three major controversies now related to the Big Five: (1) the number of basic dimensions, (2) the orthogonality of the five dimensions, and (3) the problems with overcoming the purely descriptive nature of the five-factor taxonomy of personality. Each of these problems has quite far-reaching implications for the SES model that is built within the Big Five framework.

The first problem, with respect to SES, is the question of why exactly these five basic SES domains should be distinguished to describe the diversity of SES. There are, after all, alternative models – for example, HEXACO, which distinguishes six domains, adding Honesty-Humility and reconceptualizing Agreeableness and Neuroticism (Ashton and Lee, 2007). While there are considerations on the HEXACO model in the literature on the classification of SES (Kankaraš, 2017), it is the Big Five that ultimately appears in the proposed approaches. Even if the Big Five is good enough to describe the diversity of personality traits, it is not clear why it should be optimal for describing the diversity of SES, since the nature of skills (as a malleable capacities) is different from that of traits (as tendencies; Soto et al., 2020).

Incidentally, in the first propositions to consider SES in the Big Five framework, it was believed that personality traits are also malleable and can change under the influence of external factors and learning, while SES, in contrast, demonstrate high stability (John and De Fruyt, 2015). Pellegrino and Hilton (2013), who used models of intelligence structure in addition to the Big Five to classify skills, cited research, which even demonstrated the malleability and changeability of intelligence. Such an approach, reducing the differences in malleability between traits and skills, was an important argument to justify the use of the Big Five (and intelligence) to classify and describe skills. Indeed, the definitional prerequisite was to assume malleability of skills, but – as argued – personality traits are also changeable and malleable. The integration of skills with traits in the Big Five framework is sometimes so far-reaching that, for example, Chernyshenko et al. (2018) explicitly write that “skills,” “sub-domains,” and “facets” are used interchangeably, and they review trait models not only of personality but even of temperament in order to identify facets/skills below the Big Five in the hierarchical structure of traits.

In recent years, however, the concepts of skills and traits have been increasingly distinguished. Kankaraš and Suarez-Alvarez (2019) explicitly state that they use the term skills rather than traits to indicate the possibility of change and development, and Soto (Soto et al., 2020) introduces the postulate of a necessary systematic differentiation of malleable skills from rather stable traits (see McCrae and Costa, 2003). At the same time, in both the approaches mentioned above (OECD, Kankaraš and Suarez-Alvarez, 2019 and Soto et al., 2020), SES are identified among facets of the Big Five or in another words lower-order traits. It is worth noting, however, that this means *de facto* accepting the assumption that lower-order traits of the Big Five can be divided into malleable skills and nonmalleable nonskill traits and that this can be done essentially in each of the five personality domains. This, in turn, has far-reaching theoretical implications for the Big Five models, particularly for the relationship between lower-order traits and the five basic dimensions of personality. If some lower-order traits are malleable, then the malleability of higher-order traits, that is, Big Five dimensions, becomes an issue and the distinction between malleable skills and stable traits becomes blurred again. To summarize – using the Big Five to describe SES (1) seems to be rather an arbitrary choice given competing models (e.g., HEXACO) and (2) carries quite serious theoretical implications for the Big Five itself.

The second problem of the Big Five is the orthogonality of the distinguished five basic dimensions. Orthogonality was an important thesis of the Big Five models, because it aimed to provide a set of basic, independent dimensions for describing personality. Application of the Big Five as a framework for SES in principle should also imply the adoption of the thesis that the underlying dimensions of SES are orthogonal. However, many SES models explicitly mention deviations from orthogonality. Both Soto (Soto et al., 2021) and the OECD (Kankaraš and Suarez-Alvarez, 2019) distinguish SES that are blends of Big Five traits (cf. Table 2). Similarly, of the eight main SES distinguished in the PRACTICE model (Guerra et al., 2014), half are assigned to Big Five traits and half are blends of basic traits. The far-reaching nonorthogonality of the domains is also evidenced by the Five Cs research results, in which a higher-order factor grouping of all the Cs is clearly evident (Bowers et al., 2010). It is worth noting that the question of the relationship between SES (orthogonality is a special case of such relationships) is relevant to any model or catalog of SES because it determines whether the development of SES belonging to one domain can contribute to the development of SES belonging to another domain or whether these are independent groups of SES. Knowing this is of great practical importance, yet the demand of practice is the starting point for creating SES models. Thus, the linkages and relationships between SES, grouped in the basic domains/dimensions, are crucial.

The third problem in Big Five research (a purely descriptive nature of the five-factor taxonomy) is the most significant. Applying the Big Five taxonomy to grouping and organizing SES does bring some order, but unfortunately in principle does not open up the possibility of better understanding what SES are, the mechanisms behind their development, and to locating them in the dynamic structure of personality. A model that merely assigns SES to five personality domains fails to answer a number of questions, including: (1) what are the relationships between individual SES within a domain and across domains; (2) is there any hierarchy of SES, and if so, what does it tell us – is it better to shape general SES that will extend into specific ones, or is it better to shape specific skills that shape the general one; (3) is transfer between particular skills possible; and (4) does the malleability of SES mean that they are completely undetermined by biological factors, or do people differ in some initial level of readiness to develop various SES? Lack of knowledge of the SES shaping or developmental mechanisms in the Big Five framework greatly limits the practical usefulness of such a model.

The final unresolved problem with the SES model under the Big Five framework – which is no longer inherited as a problem with the Big Five model – is the question of the optimal level of intensity of a given skill and the conditions on which that optimal level depends. Two approaches are possible, which can be provisionally named as maximalist and balanced. In the first approach, the greater the intensity of a given skill, the better – greater chances for professional and personal success, and therefore, each skill is worth strengthening almost indefinitely, regardless of other SES. In the second approach (balanced), the Aristotelian golden mean applies, so that both extreme intensities of a given dimension are suboptimal, while the optimal one is the middle one. An example is courage, located after Aristotle as the golden mean – between the wrong extremes of cowardice and bravado. Adopting the Big Five model does not allow for a conclusive answer to the question of an optimal SES intensity level. On the one hand, each of the five traits – despite the neutral nature of the description – has its adaptive pole, which together form the so-called General Factor of Personality (Musek, 2007; Rushton and Irving, 2011). This is high extraversion, agreeableness, conscientiousness, openness to experience, and emotional stability. On the other hand – there are some arguments that both extremes of a trait can be maladaptive. ICD-11 (World Health Organization, 2020), taking the Big Five as a starting point to distinguish pathological traits responsible for personality disorders, assumed that in the case of extraversion, agreeableness, and emotional stability, it is the negative pole that is maladaptive, but in the case of conscientiousness – both poles are maladaptive. They are labeled as disinhibition (extreme low conscientiousness) and anankastia (extreme high conscientiousness). This issue becomes even more pertinent given that conscientiousness is the domain that appears to be particularly important to success and well-being in the professional sphere.

## Changing the Framework From the Big Five to the Circumplex of Personality Metatraits

The SES model we propose incorporates the advantages of considering SES in the Big Five framework, while addressing the problems with the Big Five discussed above. Specifically, our proposal (1) identifies basic dimensions that match the nature of SES and that can describe diversity of SES in a relatively simple manner; (2) roots these dimensions in a complex, holistic model of personality structure with deduction of their underlying mechanisms; and (3) precisely formulates the conditions under which the maximum intensity of a given skill is optimal and under which its average intensity is optimal.

The issue of the nonorthogonality of the Big Five discussed above led to the development of the Two Factor Model of personality (review in Cieciuch and Strus, 2017). Its essence, however, is not just the reduction in dimensions, but rather the identification of the basic mechanisms underlying personality dynamics (see Digman, 1997; DeYoung, 2006, 2015). This model was later extended to the CPM (Strus et al., 2014), which distinguishes the four most basic meta-dimensions of personality (and the eight metatraits located at their poles). As we will show below, the CPM can be the basis of the SES model.

### Two Factor Model of Personality

As Cieciuch and Strus (2017) show, the TFM of personality integrates three quite different lines of psychological research. The first is the psycholexical research that originally led to the discovery of the Big Five in the English language (Goldberg, 1990), but replications in non-Germanic languages conducted since the 1990s have shown increasing problems with Big Five replicability. It is now quite widely accepted that only two broad factors appear to be fully ubiquitous across languages and cultures, and they are usually called self-regulation and dynamism (Saucier et al., 2014).

The second line of research integrated into the TFM model is questionnaire-based personality structure research, which led to the unexpected discovery of higher-order personality factors above the Big Five (Digman, 1997). One higher-order factor is formed by the shared variance of emotional stability (vs. neuroticism), conscientiousness, and agreeableness, with the other one being formed by the shared variance of extraversion and openness to experience (intellect). Digman (1997) named the former Alpha and interpreted it as a socialization factor, while the latter was named Beta and was interpreted as a personal growth factor.

The third line of research combined by TFM is the most diverse. It was initiated by Digman (1997) and includes a number of dual constructs in psychology that, in different approaches and in very different theoretical traditions, were used for describing the underlying dimensions or mechanisms that describe and explain personality or even more broadly – psychological life. Some of the best-known dual constructs that are theoretically related to the two higher-order factors of personality include the following: openness (vs. conservation) and self-transcendence (vs. self-enhancement) as basic human values (Schwartz et al., 2012); ego-resiliency and ego-control as basic properties of ego (Block and Block, 1980); power and intimacy as basic motivations (McAdams, 1988); positive and negative affect as basic dimensions of affect (Watson and Tellegen, 1985); impulsiveness (BAS) and anxiety (BIS) as basic dimensions of temperament (Gray, 1991); internalizing and externalizing problems as basic classes of psychopathology (Krueger and Markon, 2006); and also accommodation and assimilation as basic developmental processes (Piaget, 1952). It is worth noting that such dual constructs also occurred at the intersection of psychology and other sciences. In particular, a pair of concepts proposed by Bakan (1966) in philosophy were used to describe the basic modalities of human existence: Agency and Communion and a pair of concepts proposed by Grossberg (1980) in cybernetics to describe the necessary conditions for the functioning of each artificial and biological learning system: Plasticity (ability to acquire new knowledge) and Stability (ability to maintain the acquired knowledge). The Stability-Plasticity pair was used by DeYoung et al. (2002) to redefine Alpha and Beta of Digman (1997) and is commonly used nowadays to describe the two personality metatraits. Stability and Plasticity in original approach of Grossberg (1980) explicitly refer to skills, which is particularly relevant to SES, although Grossberg uses the term ability (due to the fact that he is describing a cybernetic system in which it is not possible to distinguish skills from abilities).

Two Factor Model therefore integrates: (1) the inductive discovery of two factors in psycholexical research, (2) the unexpected discovery of two metatraits in the questionnaire research on personality structure with (3) various dual constructs (mechanisms) identified in different areas of psychology and beyond. This means that the constructs highlighted in the TFM are not merely dimensions that only combine descriptive traits, but have great theoretical potential to explain the entire personality functioning.

### Circumplex of Personality Metatraits

**Figure 3:**
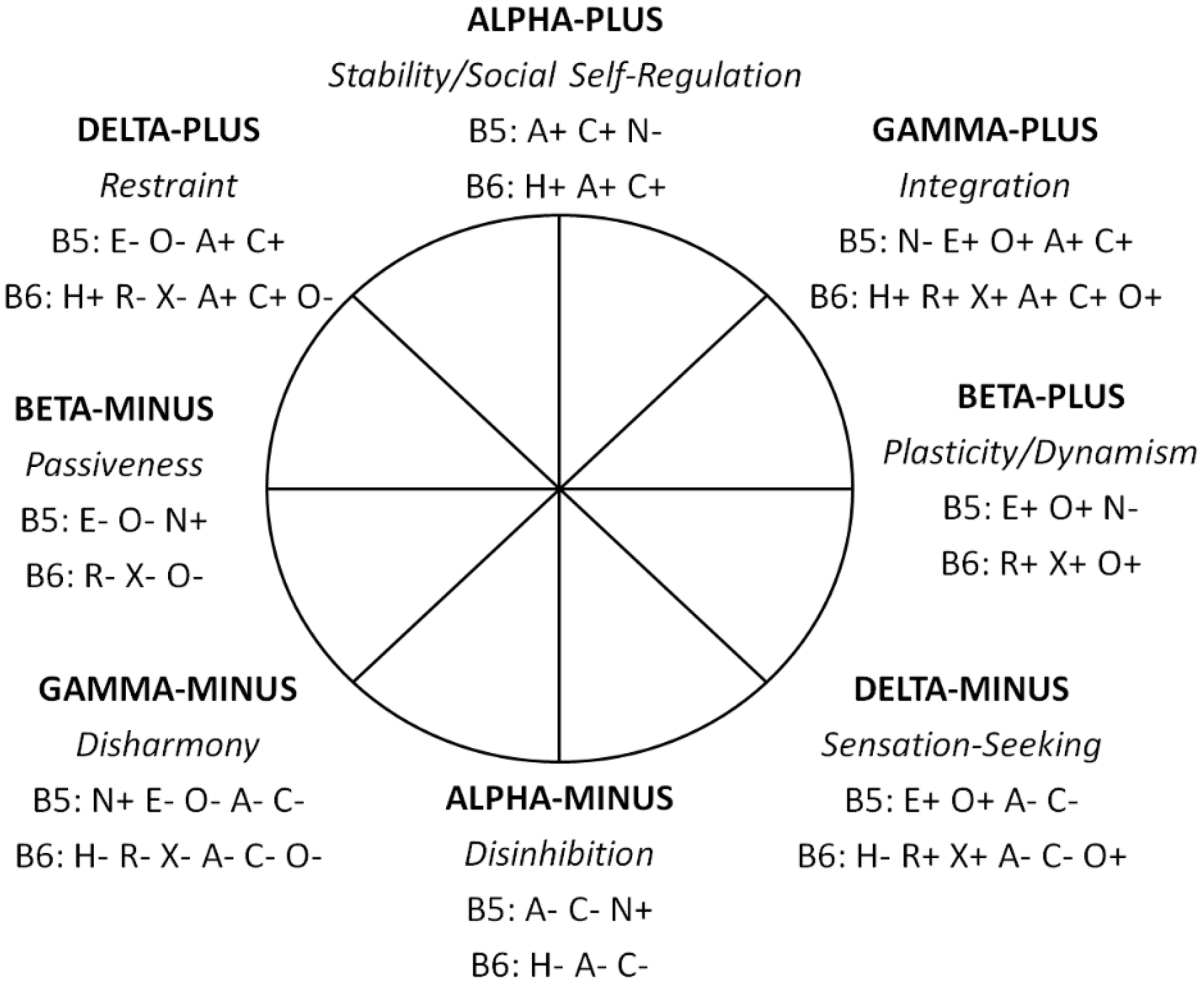
The Big Five and Big Six traits within the modified Circumplex of Personality Metatraits. B5, Big Five traits; N, Neuroticism/Emotional Stability; E, Extraversion; O, Openness to experience/Intellect; A, Agreeableness; C, Conscientiousness; B6, Big Six traits; H, Honesty-Humility/Propriety; R, Resiliency/Emotionality; X, Extraversion; A, Agreeableness; C, Conscientiousness; O, Originality/Openness to experience; + positive pole of the trait; − negative pole of the trait (Strus and Cieciuch, 2021).

Circumplex of Personality Metatraits proposed by Strus and colleagues (Strus et al., 2014; Strus and Cieciuch, 2017, 2021) continues the line of thinking in terms of broad personality dimensions. The CPM applies the idea of circular organization of metatraits, arranging Alpha/Stability and Beta/Plasticity as orthogonal axes within a circumplex structure. In addition, the CPM incorporates two other metatraits, that is, Gamma/Integration and Delta/Restraint, which are located orthogonally to each other and at a 45 degree rotation to the Alpha/Stability and Beta/Plasticity. Importantly, the CPM in its refined version (Strus and Cieciuch, 2021) defines Alpha/Stability and Beta/Plasticity not only in terms of the five factors of the FFM, but also by using the six factors of the HEXACO, while somewhat reconstructing the metatraits built over the Big Five. The model is presented in Figure 3, while the metatrait definitions can be found in Table 3.

**Table 3:** Description of the eight metatraits in the revised Circumplex of Personality Metatraits.

Metatrait	Meaning
Delta-Plus (Restraint)	Low emotionality (both negative and positive), high behavioral and emotional control, meticulousness, and perfectionistic tendencies as well as modesty, conventionality, and severe social adjustment.
Alpha-Plus (Stability)	Stability in the area of emotional, motivational, and social functioning, expressed as a general social adaptation tendency, an ethical attitude toward the world, benevolence, and calmness, as well as the ability to delay gratification, diligence and perseverance.
Gamma-Plus (Integration)	Well-being, a warm and prosocial attitude toward people, both intra- and interpersonal balance and harmony; serenity, openness to the world in all its richness, as well as endurance and effectiveness in attaining important goals.
Beta-Plus (Plasticity)	Cognitive and behavioral openness to change and engagement to new experiences, a tendency to explore, self-confidence, initiative and invention in social relations, enthusiasm and orientation toward personal growth.
Delta-Minus (Sensation-Seeking)	Broadly defined impulsiveness, recklessness, emotional volatility, stimulation seeking and risk taking; self-enhancement and hedonistic tendencies as well as interpersonal dominance and expansiveness.
Alpha-Minus (Disinhibition)	High level of antisocial tendencies underpinned by unsustainability, low frustration tolerance and egotism as well as aggression and antagonism toward people, social norms, and obligations.
Gamma-Minus (Disharmony)	Inaccessibility, coldness and distrust in interpersonal relations; negative affectivity and low self-worthiness; depressiveness, pessimism and proneness to suffer from psychological problems.
Beta-Minus (Passiveness)	Social avoidance and timidity, along with submissiveness and dependency in close relationships; cognitive and behavioral passivity and inhibition; some type of stagnation, apathy, and tendency for anhedonia.

Inheriting, as it were, the integrative potential of the TFM and further extending it by identifying two additional metatraits, the CPM has become a general model of personality, synthesizing several models of various personality variables. Previous empirical research supports this synthesizing potential of CPM, which (1) integrates the Big Five and HEXACO (Strus and Cieciuch, 2021); (2) allows for demonstrating subtle differences between the Big Two derived from the psycholexical and questionnaire traditions (Strus and Cieciuch, 2019); (3) allows for the integration of models of temperament, emotion, motivation, values, well-being, and mental health problems, including personality disorders, into a single framework (Strus and Cieciuch, 2017; Zawadzki, 2017; Rogoza et al., 2019). Moreover, as demonstrated by Strus et al. (2021c); (4) the CPM has proved to be useful for resolving which pathological Big Five is more justified – the one proposed by the DSM-5 (American Psychiatric Association, 2013) or by the ICD-11 (World Health Organization, 2020); and (5) it has helped resolve issues with the number and content of personality types (Strus et al., 2021a,b). The CPM is also used to create new models in which the relationships between constructs are precisely defined in a reference system of two basic dimensions, as in the CPM. This was the case for the example of (6) Rogoza et al. (2021, under review) who constructed a model of vulnerable narcissism in this way, (7) Rymarczyk et al. (2020) who proposed a reconceptualization of type C of personality, and (8) Strus et al. (2021, under review) who developed a new model of temperament. Given the above, the CPM seems a good candidate to be used for developing the SES model.

### Terminological Clarifications: Personality Competencies and Social-Motivational-Emotional Skills

In the literature on SES, the terms *skill* and *competence* are often used interchangeably. This is the case both in the literature from the area of basic and applied research highlighted above, as well as in official documents. The name of the *21st century skills* construct contains the *skills* term, while in the systematizing proposal of Pellegrino and Hilton (2013), 21st century skills are divided into three domains of competencies. Similarly, the term *key competencies* used in documents of the European Union basically refers to 21st century skills. In their comprehensive compilation of multiple SES models and related constructs, Berg et al. (2017) also use the term *competence*. We argue, however, that these terms, although used interchangeably, are worth distinguishing because they refer to slightly different subjects. Below we suggest how skills can be systematically distinguished from competencies.

In the models proposed by the OECD (Kankaraš and Suarez-Alvarez, 2019) and especially Soto and colleagues (Soto et al., 2020, 2021), the term *skills* is used precisely and is clearly distinguished from traits (as a reminder, skills mean capacity, and traits mean tendency). This approach has the advantage of being unambiguous and precise, but reducing skills to pure capacity is problematic. To see the problem, let us consider empathy as an example. Empathy can be understood as the capacity to understand other people’s thoughts and feelings. However, a skill understood in this way does not mean that empathic behavior will occur and that a person, who has such a capacity, will demonstrate an understanding of another person’s feelings and thoughts even if there is an opportunity for that. This is because capacity is only the possibility (potential), and there still needs to be a motivational element that triggers the behavior. But where does this element belong – to skills, traits, or somewhere else? It seems that it is the term *competence* that contains such an element that actualizes the potential of skill (understood as capacity). Skill is just a capacity that may or may not be activated in behavior. Competence, on the other hand, includes motivational elements in addition to skill-specific capacity. The Council of the European Union (2018) defines competence as a combination of skill, knowledge and attitude, which also captures this idea of competence that is realized in the appropriate way under the given conditions (knowledge and attitude).

Previous approaches to systematize SES started with specific skills and consisted of grouping them into the Big Five domains. It was an approach analogous to those that led to the discovery of the Big Five itself. In our proposed approach to SES through the lens of the CPM, the case is different. This is because the starting point here is not specific skills, but looking for general characteristics necessary for satisfactory and effective functioning. The category of competence seems to be particularly useful here because by containing some additional motivational elements, it is broader than skill. Moreover, these additional motivational elements also additionally makes it possible that a competence can be composed by a set of several detailed skills.

This understanding is also supported by linguistic intuitions – both contemporary and etymological. Let us start with the former. When we attribute competence to someone, the implication is that this person (a) uses his or her skills in a way he or she behaves (b) also in situations he or she has never been in and which may in fact require some new skills. For example, a competent teacher is one who not only knows how to teach, but really does so effectively. He or she therefore has and applies skills of attracting student interest, disciplining students, conflict resolution, but also perhaps of working on Self-motivation and preventing professional burnout. Moreover, he or she is able to operate in both routine and nonroutine teaching situations he or she has never found himself or herself in before, using various specific SES and other skills he or she possesses. This distinction between specific skills and broader personal competence is also consistent with the etymology of the words “skill” and “competence.” The etymological root of the word ‘skill’ led to old the Norse and Proto-Germanic meaning of “difference”, while the etymological root of the word “competence” led to the Latin meaning of “meeting together, agreement” (Online etymology dictionary at https://www.etymonline.com on 04/02/2021); thus, etymologically, skill is related to “differentiation” while competence to “synthesizing.”

In our model, we adopt the distinction between traits and skills proposed by Soto (Soto et al., 2020), but we focus on basic competencies, which include various SES, combined with knowledge and attitudes and thus mean applying the skills in real behavior. One could say that the relationship between skills and competencies is analogous to the relationship between traits and metatraits in the CPM when interpreting metatraits as basic dispositions or mechanisms underlying traits rather than just constellations of traits.

In the case of competencies that underlie and organize SES, we propose the label of *personality competencies*. One of the definitional features of SES was their association with positive outcomes in various life spheres, including socioeconomic outcomes (e.g., John and De Fruyt, 2015). Moving from the level of specific SES to the level of general personality competencies, it is also worth generalizing the usefulness of these outcomes – from many detailed outcomes to social, personal, and vocational well-being. Incidentally, well-being, as a generalization of positive outcomes, already appears in the literature (Chernyshenko et al., 2018), so we continue this line of thinking.

Following Soto’s proposal (Soto et al., 2020, 2021) to include a broader range of skills under this label and in order to distinguish between two different meanings of the SES acronym, namely, “social and emotional skills” and “socioeconomic status,” whenever we talk about our proposal, we will use the term SEMS – that is, social, emotional and motivational skills, because the ability to motivate oneself is an important domain of SES, which often appears in various SES catalogs.

We therefore formulate the following definitions: Personality competencies (PC) are consistent patterns of thoughts, feelings, and behaviors that (a) enhance well-being in various life domains including work, personal and social life, (b) can be developed through formal and informal learning experiences, and (c) underlie a number of specific SEMS. In turn, SEMS can be defined after Soto et al. (2021) as a capacity to maintain mutually satisfactory social relationships, regulate impulses and emotions, and manage goal-directed behaviors.

## Toward a Model of Personality Competencies Within the Framework of Circumplex of Personality Metatraits

Below, we propose the model of basic PC, that can be shaped in education and are necessary for human well-being. In order to identify such competencies, we follow two ways that are analogous to two sources of SES catalogs developed so far in the literature: (1) The first is to identify people’s basic characteristics necessary for sound functioning in society, effective work or stated at general level – for overall well-being, and (2) the second is to identify basic competencies in an established model of personality structure that is analogous to the procedure adopted by Soto (Soto et al., 2020, 2021) or the OECD (Kankaraš and Suarez-Alvarez, 2019), with the CPM rather than the Big Five as the reference model. As we show below, both ways lead to the same PC catalog.

### The First Way – A Catalog of Personality Competencies Enhancing Well-Being

*Human beings act in a social context* – such a statement is a truism that is hard to disagree with. At the same time this obvious statement can be a good starting point for constructing the most general PC catalog. According to this brief statement, human activity takes place in two domains, which could be referred to as a task domain (*human acts*) and a social domain (*in a social context*). Of course, these two domains intersect; nevertheless, the realms of action and context are distinguishable. The SES domains differentiated by John and De Fruyt (2015) are close to this division: Achieving goals is the task domain, and Working with others is the social one, while Managing emotions combines both domains. Also, the distinction between intraindividual and interindividual domains that appear in many places in the literature (American Psychiatric Association, 2013; Pellegrino and Hilton, 2013; Domitrovich et al., 2017) is a similar distinction, albeit not identical.

The essence of human activity, then, is an action that is purposeful (the task domain) and takes place in a world that is largely a social world (the social domain). Thus, it can be said that people need such PC that enable them to (1) take effective purposeful action and (2) function well in social relationships. The question then arises – what types of PC are these? The most general answer is: In terms of taking effective purposeful action, one needs: (1a) Self-motivation competency to strengthen own (his/her) intentions, goals, motivations, and (1b) Impulse control competency to appropriately control impulse, urges and affective reactions that may interfere with performing the action. In the domain of social relationships, both of the following are needed: (2a) Social responsibility competency, to be able to enter into communion and make mutually satisfying relationships with others, and (2b) Assertiveness competency to be able to maintain one’s autonomy and agency while entering into social relationships. This results in four competencies that, albeit at a general level, describe social-emotional-motivational functioning in a comprehensive way.

### The Second Way – A Catalog of Personality Competencies Identified Within the Circumplex of Personality Metatraits

The CPM model describes personality functioning in terms of metatraits, distributed on the circumplex that is constituted by two orthogonal dimensions: Alpha/Stability and Beta/Plasticity. Stability and Plasticity are the two mechanisms whose proper functioning is responsible for sound functioning, mental health, and well-being. This means that core PC that contribute to sound functioning and well-being can be identified and located at the positive Alpha and Beta poles (Alpha-Plus/Stability and Beta-Plus/Plasticity, respectively). Such competencies can also be located at the positive pole of Gamma (Gamma-Plus/Integration), because Gamma-Plus is related to high intensity of Alpha-Plus and high intensity of Beta-Plus. The positive poles of the dimensions listed above describe the competencies responsible for effective functioning, mental health, and well-being, which means that the higher the intensity of the competencies located therein, the higher the well-being. The case is different for the Delta dimension (see Figure 3). This is the line that separates healthy functioning (above the Delta line) from potential problems (below the Delta line; for details, see Strus et al., 2021c). In the case of Delta-Plus, the intensity of Stability is still high, while that of Plasticity is low. Thus, it could be said that functioning is based on only one mechanism (Stability) with a deficit of the other (Plasticity). This is therefore a border and potentially dangerous situation – a further decrease in Plasticity may mean that Stability is no longer enough to ensure sound functioning. The analogy is Delta-Minus, in the case of which healthy functioning is based on only one mechanism, Plasticity (with a deficit of Stability). A further decrease in the intensity of Stability may lead below the Delta line and therefore to the area of problems with sound functioning and well-being. This structure has far-reaching implications for PC. All competencies located in Alpha-Plus, Gamma-Plus, and Beta-Plus are desirable in the sense that their increase always contributes to improved personal and social functioning. The case is different for competencies located in Delta-Plus and Delta-Minus. Their extreme intensity combined with weak Stability and/or Plasticity weakens effective functioning and well-being, while its medium intensity promotes it.

The question is what exactly PC are located in high intensity Alpha-Plus/Stability, Beta-Plus/Plasticity Gamma-Plus/Integration, and located between high intensity of Delta-Plus/Restraint and Delta-Minus/Sensation seeking? These seem to be competencies that show a far-reaching convergence with those distinguished in the previous paragraph.

The important element of the meaning of Delta is emotional and behavioral control vs. impulsiveness and risk taking (see Table 3). In turn, Gamma-Plus seems to be the center of effectiveness in attaining important goals. Therefore, Gamma and Delta can be treated as the theoretical basis for two fundamental self-regulation competencies in the task domain, that is, Self-motivation and Impulse control, respectively. On the other hand, in the personality competence context, Alpha and Beta can be deemed as mainly concerning the social functioning domain, as these metatraits strongly correspond to constructs of Communion and Agency, respectively, which are often used especially in social psychology (Abele and Wojciszke, 2014). In consequence, Alpha – as a socialization and communion factor – can be treated as a basis for Social responsibility competence; in turn, Beta – as a personal growth and agentic factor – can be treated as a basis for Assertiveness competence (see Digman, 1997). These competencies will be described below.

#### Self-Regulatory Personality Competencies in the Task Domain

Self-regulatory processes take place both in relation to intentionally undertaken and realized goals or intentions and in relation to automatically or involuntarily aroused drive-affective impulses. Accordingly, the emotional-motivational self-regulation system contains two distinct and essentially independent mechanisms: Self-motivation and Impulse control, while the effectiveness of these mechanisms in a given individual reflects a certain level of that individual’s self-regulatory (emotional-motivational) competencies. These competencies are thus expressed in the ability to manage and direct emotional-motivational processes (intentions and impulses; see Block and Block, 1980; Bandura, 1989; Kuhl, 1992).

**Self-motivation** is therefore a competence that is the basis for the capacity to strengthen motives related to the attainment of broadly defined goals and intentions, for example, values, personal standards, or commitments. These motives tend to be cognitively advanced structures that, from a motivational standpoint, tend to be weaker and more fragile than drive-emotional impulses. In order to motivate behavior, these structures must obtain the person’s engagement, which then can either fade away or be fueled and sustained. Therefore, intention reinforcement can take place at three different time-points of the process, that is, in a phase of: (1) making the decision and triggering its implementation (initiating the activity); (2) carrying out the activity (sustaining the engagement); and (3) completing the activity (evaluating its effects) and being able to undertake the next activity.

**Impulse control** is the competence to regulate impulsive behavior. Drive-emotional impulses occur essentially independently of the person’s will and intention, and they can be initiated from within (e.g., an organism’s need) or from without (an external stimulus). In terms of functionality at the dispositional level, both opposite poles of impulse control, that is, both permanent impulse inhibition (as a result of an overactive control mechanism) and impulsiveness (as a result of an underactive control mechanism) are maladaptive. The adaptive form of impulse control, on the other hand, is the capacity to both inhibit impulses and realize them depending on the actual external (current circumstances) and internal situation (e.g., currently realized action) as well as in an appropriate form. The competence associated with the sound functioning of the mechanism described is therefore the capacity for flexible and controlled realization (expression) of impulses in an adequate manner.

The two competencies, although independent, often operate at the same time, and behavior is frequently the result of their interaction. For example, in pursuing a goal, Impulse control is responsible for weakening competing motives, while Self-motivation is responsible for strengthening the very intention to attain the goal.

#### Interpersonal Personality Competencies in the Social Domain

We treat the competencies of Social responsibility and Assertiveness as an expression of proper functioning of two basic mechanisms regulating social life of human beings: entering into relations with others and maintaining own individuality and autonomy, respectively.

In **Social responsibility**, community, other people, and the individual’s relationship with them play a key role, and this competence is formed in the course of social development, inclusion in the group and the process of an individual becoming an integral part of society. Thus, Social responsibility can be understood as the capacity to anticipate and take into account the consequences of one’s behavior for other people, to understand the internal states of others and respond emotionally to their situation, to identify with a social group and have a sense of being an integral part of some broader whole, as well as be guided in behavior by an internalized system of moral and social norms.

In the case of **Assertiveness**, the individual himself or herself as well as his or her needs realized in the social environment are of key importance, and this competence is formed in the course of separation and strengthening of the individual’s self, and shaping his/her autonomy, subjectivity, and agency. Assertiveness competence is therefore built on the foundation of stable self-esteem and is connected with the capacity to perceive oneself positively and at the same time adequately regardless of current events, with confidence in one’s own abilities and a strong conviction that one is a person who can effectively influence his or her surroundings and deal with adversities, as well as with the capacity to influence other people and function effectively in a group.

## Summary of Advantages and Possibilities of the Proposed Model of Personality Competencies

The identification of core PC within the CPM has several advantages. The most important of these are listed below.

**Figure 4:**
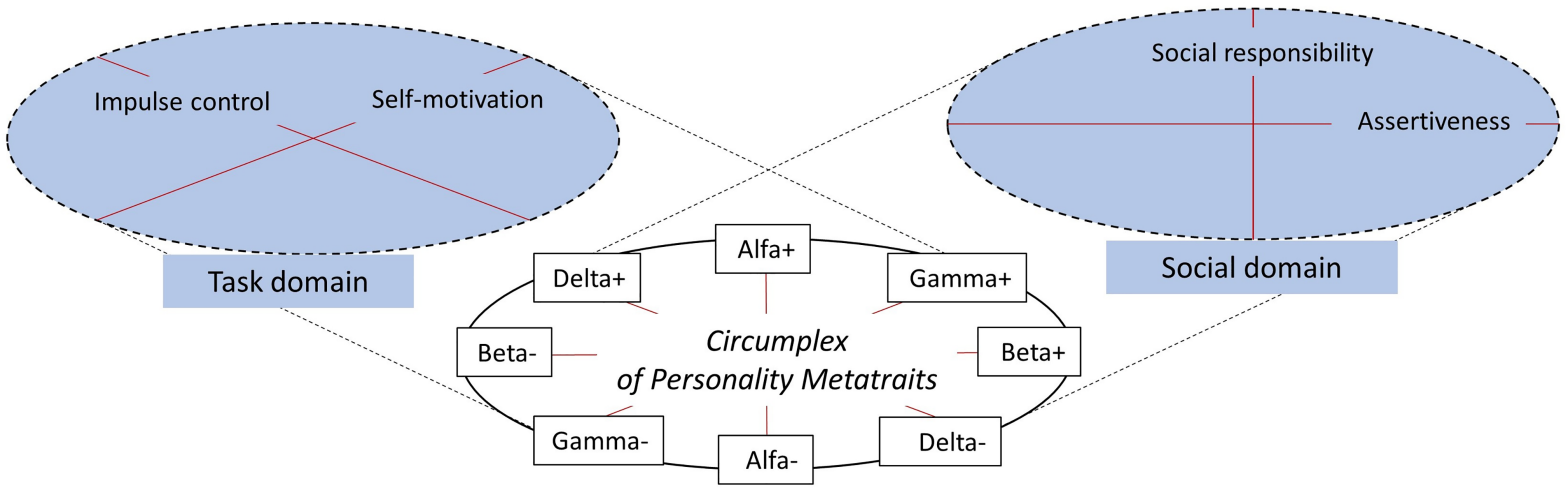
Personality competencies within the Circumplex of Personality Metatraits.

First, we can assume that our proposed model identifies all the key PC because they were found in a general model of personality structure. Moreover, the relationships between the distinguished PC are precisely defined. Social responsibility and Assertiveness are orthogonal to each other and correspond to the Alpha and Beta dimensions in the CPM. Also, Self-motivation and Impulse control are orthogonal to each other and correspond to Gamma and Delta in the CPM. Therefore, Self-motivation and Impulse control are shifted 45 degrees in relation to the Social responsibility and Assertiveness arrangement. These relationships are shown in Figure 4.

Within a given competency, many specific SEMS can be distinguished. It is also possible to find and define skills that combine various PC, with the relationships and contributions of a given general PC to a specific skill following the relationships described above and presented in Figure 4. Thus, one can say that the PC we have distinguished provide a kind of matrix for locating many specific SEMS. What is more, the SES distinguished in the various catalogs discussed above are definable by the PC we have distinguished, although showing this is beyond the scope of this paper.

Second, adopting the CPM as a framework for PC and SEMS allows us to distinguish malleable SEMS from enduring traits, which should be treated, however, as temperamental traits instead of personality traits. A temperament model was also constructed within the CPM framework, in which two basic orthogonal dimensions of Reactivity and Activity were distinguished (Strus et al. 2021, under review). Certain configurations of given temperament traits facilitate the acquisition of certain PC, while others hinder it. Knowledge of temperamental conditions allows interventions to be tailored to optimize the development of PC.

Third, the CPM framework allowed precise determination of the conditions under which the maximum intensity of a given SEMS is optimal and under which the average intensity is optimal. Maximum intensity is optimal for all SEMS lying above the Delta line, namely, SEMS rooted in Self-motivation, Social responsibility, and Assertiveness. Average intensity, on the other hand, is optimal for the Delta line; therefore, for all SEMS rooted in the general ability to realize impulses in a controlled (adequate) manner (Impulse control), the Aristotelian golden rule applies of mean between inhibition and impulsiveness.

Fourth, the PC model identified in the CPM framework is not just a descriptive taxonomy, as it allows the identification of key mechanisms important to personal and professional sound functioning and well-being. From this point of view, it seems more fruitful to focus in education on developing the core PC – especially knowing their underlying mechanisms – rather than specific SEMS, because PC are a kind of reservoir from which various SEMS can grow.

**Figure 5:**
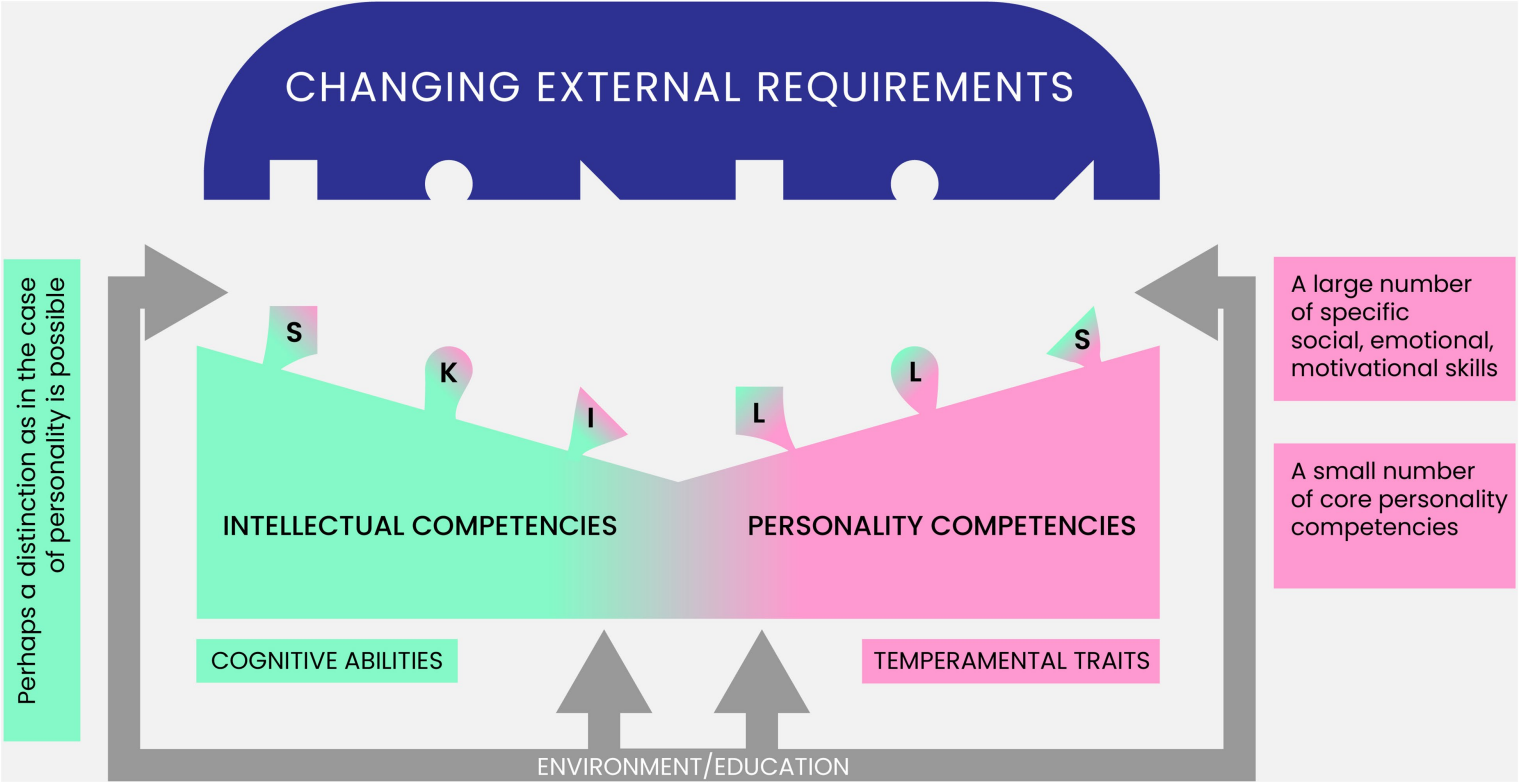
A comprehensive model of competencies and skills both in cognitive and noncognitive domains.

To summarize, we propose a comprehensive model of (a) malleable core PC that, on the one hand, (b) are determined by stable, biologically based temperamental traits and, on the other hand, (c) underlie many specific SEMS. Such a holistic model is presented in Figure 5.

Specific SEMS are tailored to external changing demands and grow precisely out of the reservoir of PC. Environmental interventions, including education, can be directed toward shaping specific SEMS, but also toward shaping core PC, especially compensating for stable nonoptimal configurations of temperamental traits. However, it is the focus on shaping core competencies that seems to be a more effective solution, particularly in light of recognition of their underlying mechanism that we proposed. The model presented in Figure 5 also considers the cognitive domain. The counterpart of enduring temperament traits are cognitive abilities. It may be worthwhile in the future to consider describing intellectual competencies in an way that is analogous to that we have described personality competencies herein.

## Limitations and Further Directions

The presented model is based on the thorough literature review and built on the current knowledge on personality structure and socio-emotional functioning. However, although this model can be deemed as theoretically justified, it has not yet been empirically verified, which should be admitted as a main limitation of this proposal. In order to overcome this limitation, the following four-step research agenda is proposed.

In the first step, a more detailed conceptualization of the model is needed. For now, four main PCs are differentiated; however, there are many unknowns in this respect. In particular, what are the components or facets of these PC? Is it possible and desirable to distinguish such facets in order to fully and precisely cover the theoretical meaning of these constructs? Next, what are the mechanisms underlying the PC. Although these mechanisms were initially outlined above, they should be further elaborated, as it seems that their precise identification is necessary for practical application of the model in order to help in developing these PCs during intervention and education.

In the second step, the differentiated PC and/or their facets have to be operationalized. The measurement instruments should be prepared for both self- and other-report. Moreover, the instruments should be designed for various developmental or educational periods because the PC are shaped during education to a larger extent.

In the third step, the model proposed above should be empirically verified. Especially important are the relations between (1) PC and detailed SEMS that are rooted in PC, (2) PC and metatraits from the CPM, that organize the structure of PC, and (3) PC and temperamental nonmalleable traits that determine the susceptibility for development of PC and can help to make interventions more effective.

In the fourth step, the usefulness of the proposed model in practical (e.g., educational) settings should be examined and evaluated. Particularly, interventions to enhance the developing PC must be proposed and their effectiveness should be measured with a rigorous research design. Of particular importance is testing the effectiveness of developing general PC for shaping the detailed SEMS.

Therefore, although the paper presented the theoretical foundation and outlined the theoretical “heart” of the new model of PC (and SEMS), further efforts and research on the model’s conceptualization, operationalization, verification, and application are needed.

## Author Contributions

JC: literature review and a draft of the paper. JC and WS: conceptualization of the model and final version of the paper. All authors contributed to the article and approved the submitted version.

## Funding

European Union through the European Social Fund under the Operational Programme Knowledge Education Development 2014–2020. Project: Development and dissemination of diagnostic instruments supporting psychological and pedagogical service - emotional and social domain, POWR.02.10.00-00-9004/17.

## Conflict of Interest

The authors declare that the research was conducted in the absence of any commercial or financial relationships that could be construed as a potential conflict of interest.

## Publisher’s Note

All claims expressed in this article are solely those of the authors and do not necessarily represent those of their affiliated organizations, or those of the publisher, the editors and the reviewers. Any product that may be evaluated in this article, or claim that may be made by its manufacturer, is not guaranteed or endorsed by the publisher.

## Footnotes

^1^ www.battelleforkids.org
